# A psychospiritual approach to the integration of Rohingya refugees in Aceh: overcoming stigma and building solidarity

**DOI:** 10.1017/S0033291724002150

**Published:** 2024-10

**Authors:** Rizky Andana Pohan

**Affiliations:** 1 Department of Islamic Guidance and Counseling, Institut Agama Islam Negeri Langsa, Langsa, Indonesia; 2 Department of Guidance and Counseling, Universitas Negeri Malang, Malang, Indonesia

Dear Editor,

I am writing to highlight the mental health and social integration issues faced by Rohingya refugees who recently arrived in Aceh. The community rejection and stigma they face mirrors the findings in a recent study by Anderson et al. (2024) on the role of language differences in the risk of psychotic disorders among migrant groups (Anderson et al., 2024). This study suggests that linguistic distance may influence the risk of psychotic disorders, although the effect is small. However, the implications of these findings are crucial for the management of refugees around the world.

Rohingya refugees, like other migrant groups, also face significant language and cultural barriers that may prevent them from seeking and receiving adequate mental health care (Haider, Maheen, Ansari, & Stolley, 2023). This research shows that these barriers not only increase the risk of psychotic disorders but also hinder overall mental wellbeing (Sudheer & Banerjee, 2021). In Aceh, these barriers are exacerbated by community stigma and rejection, further isolating refugees and exacerbating their mental health problems.

Sociologically, Rohingya refugees and the Acehnese community share a common religious background, Islam. This religious attachment should provide a strong basis for building solidarity and empathy between refugees and the local community (Sadjad, 2022). However, previous bad experiences, which may involve social and economic issues, have made them start to reject the arrival of Rohingya refugees. This rejection, while understandable from the point of view of past negative experiences, certainly goes against the values of solidarity and humanitarian aid.

To address these challenges, it is crucial to consider a psychospiritual approach that integrates mental health support with spiritual care. This approach can capitalize on the strong religious and cultural ties within the Rohingya community and the host community in Aceh, bridging the gap created by language and cultural differences. A psychospiritual approach involves psychological counseling combined with spiritual guidance, providing a holistic approach that addresses both the emotional and spiritual needs of the individual (Sperry, 2013; Winkeljohn Black & Klinger, 2022). For Rohingya refugees, this can include culturally sensitive counseling, where mental health services are delivered by professionals who understand the Rohingya's cultural and religious background, ensuring that treatment is respectful and relevant (Herman et al., 2007; Zakaria & Mat Akhir, 2017). Community support programs are also crucial, involving local religious leaders and community members in providing support and reducing stigma, including joint prayer sessions, religious education, and cultural exchange programs to promote understanding and acceptance (Ilyas, 2020; Pohan et al., 2024a, 2024b). Language and integration support, such as language classes and integration programs, can help refugees navigate their new environment more effectively, reducing linguistic barriers that contribute to mental health issues.

Experience from different parts of the world shows that psychospiritual approaches have been effective in supporting refugees' mental health. For example, in Uganda, psychospiritual approaches were used to support refugees from the Democratic Republic of Congo, who faced the trauma of war and violence (Kiyala, 2019). In Lebanon, a psychospiritual programmed was implemented for Syrian refugees, who showed significant improvements in their mental well-being and social integration (Winiger & Goodwin, 2023). Therefore, by creating a supportive community environment and reducing stigma through culturally and religiously sensitive practices, we can help reduce mental health risks and facilitate better integration for Rohingya refugees in Aceh.
